# The Versatile Biocatalyst of Cytochrome P450 CYP102A1: Structure, Function, and Engineering

**DOI:** 10.3390/molecules28145353

**Published:** 2023-07-12

**Authors:** Yudong Sun, Xiaoqiang Huang, Yoichi Osawa, Yuqing Eugene Chen, Haoming Zhang

**Affiliations:** 1 Department of Pharmacology, University of Michigan, Ann Arbor, MI 48109, USA; yudons@umich.edu (Y.S.); osawa@umich.edu (Y.O.); 2 Department of Internal Medicine, University of Michigan, Ann Arbor, MI 48109, USA; echenum@umich.edu

**Keywords:** CYP102A1, directed evolution, computational protein design, machine learning

## Abstract

Wild-type cytochrome P450 CYP102A1 from *Bacillus megaterium* is a highly efficient monooxygenase for the oxidation of long-chain fatty acids. The unique features of CYP102A1, such as high catalytic activity, expression yield, regio- and stereoselectivity, and self-sufficiency in electron transfer as a fusion protein, afford the requirements for an ideal biocatalyst. In the past three decades, remarkable progress has been made in engineering CYP102A1 for applications in drug discovery, biosynthesis, and biotechnology. The repertoire of engineered CYP102A1 variants has grown tremendously, whereas the substrate repertoire is avalanched to encompass alkanes, alkenes, aromatics, organic solvents, pharmaceuticals, drugs, and many more. In this article, we highlight the major advances in the past five years in our understanding of the structure and function of CYP102A1 and the methodologies used to engineer CYP102A1 for novel applications. The objective is to provide a succinct review of the latest developments with reference to the body of CYP102A1-related literature.

## 1. Introduction

Cytochrome P450 (CYP or P450) is a superfamily of heme-containing enzymes that have been extensively studied for over five decades. A large number of CYP genes have been found in all kingdoms of life, including 57 in humans, 1056 in mammals, 3753 in bacteria, and many more in plants and fungi [1]. P450s utilize electrons from NAD(P)H to catalyze oxygen insertion into inert C–H bonds, overcoming a high activation energy barrier. P450-catalyzed reactions were first reported in 1955 [2]; it was found that tissue catalysts in the liver microsomes of rabbits were responsible for the metabolism of multiple foreign compounds like hexobarbital and ephedrine. In 1958, Klingenberg characterized and named the catalyst P450 because he discovered the emergence of absorption at 450 nm from rat liver microsomes upon the addition of carbon monoxide (CO) [3]. In 1963, the seminal work by Omura and Sato identified P450s to be a heme-containing protein [4]. Human P450s play an important role in the metabolism of drugs, biopharmaceutical reagents, toxins, and environmental pollutants [5]. The metabolism of drugs by P450s leads to drug metabolites, which are more hydrophilic and are excreted more easily. In bacteria and fungi, P450s are involved in both anabolic and catabolic processes, such as antibiotic synthesis and degradation.

P450-catalyzed reactions depend on electron transfer (ET) from NAD(P)H via redox partners to split dioxygen and insert a single oxygen atom into the substrates [6,7]. The redox partner of mammalian hepatic P450s is NADPH-cytochrome P450 oxidoreductase (POR). POR is a diflavinal protein containing one flavin mononucleotide (FMN) and one flavin adenine dinucleotide (FAD) as its cofactors, capable of sequentially transferring two electrons from NADPH to the heme moiety of P450s where monooxygenation occurs. The center of all P450s is a prosthetic heme where the heme Fe is ligated to the four pyrrole rings of protoporphyrin IX, forming the heme plane to a cysteinyl residue forming the axial thiolate ligand, as in chloroperoxidase and nitric oxide synthetase. The unique thiolate-Fe ligand produces a signature absorption peak at 450 nm when CO binds to the ferrous heme, Fe^2+^. At the resting state (Fe^3+^), the sixth axial ligand is usually occupied by a weakly bound water molecule, resulting in a low-spin ferric heme [8,9]. When the water ligand is expelled by substrate molecules, the heme transits from a low- to high-spin state, a useful feature widely used for spectral titration to determine the substrate binding constant.

**Figure 1:**
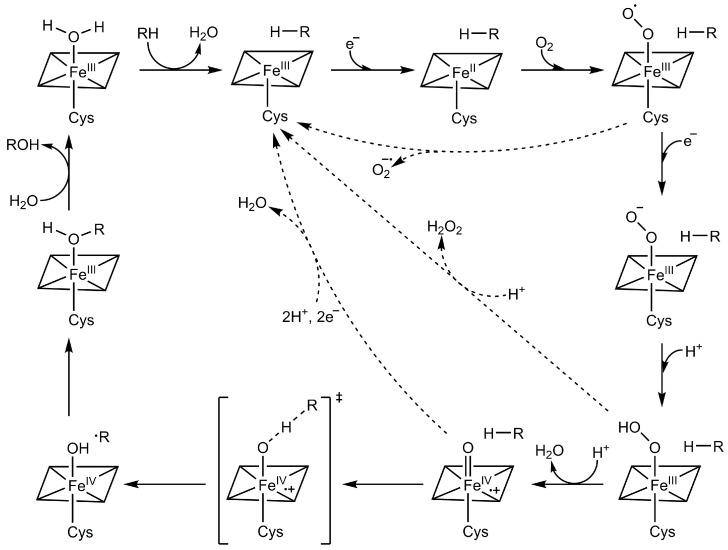
P450 catalytic cycle. RH, substrate; Cys, thiolate ligand. The three dashed lines indicate potential routes of uncoupling reactions leading to the formation of byproducts like superoxide and hydrogen peroxide.

P450s catalyze the oxidation of a vast number of small molecules, estimated to be in the range of millions. The catalytic prowess of P450s can be attributed to their ability to accumulate oxidizing equivalents through the catalytic cycle (Figure 1). As aforementioned, substate binding dissociates the sixth aquo ligand, increasing the redox potential of the heme that facilitates ET [10,11]. Then, an electron from NADPH is transferred to the heme, reducing the ferric iron to the ferrous state (the first electron), followed by rapid binding of dioxygen to the heme forming the oxyferrous species. Subsequent delivery of the second electron and two protons through proton relay machinery leads to the formation of a highly oxidizing intermediate, referred to as Compound I. P450 Compound I is a Fe(IV)-oxo species with an additional oxidizing equivalent delocalized in the thiolate-heme moiety [12]. Through a proton abstraction and rebound mechanism originally postulated by Groves [13], a single oxygen atom is inserted into the C–H bond, forming hydroxylated products. Dissociation of the hydroxylated product from the active site returns the catalytic cycle to the resting ferric state. The redox potential of P450 Compound I is estimated to be >1 V [14]. As such, P450 is nicknamed mother nature’s “blow torch”. An unwanted feature of the catalytic cycle is the formation of byproducts, such as superoxide and hydrogen peroxide, via uncoupling reactions (Figure 1, dashed arrows). The formation of these byproducts not only reduces the catalytic coupling efficiency (the molar ratio of the product/NADPH) but also increases the risk of oxidative stress in vivo.

The extraordinary capability of P450s to activate inert C–H bonds at ambient temperatures has attractive applications in biosynthesis, drug discovery, and bioremediation [15,16]. Furthermore, the P450-catalyzed reaction is regio- and stereoselective. These desirable properties of P450s appeal to synthetic chemists because conventional chemical synthesis requires harsh conditions to activate inert C–H bonds, often with low yield and poor regio- and stereoselectivity. The synthesis of drug metabolites was incentivized after the Federal Drug Administration (FDA) issued guidance on the safety testing of drug metabolites in 2016. According to the latest guidance issued in March 2020, disproportionate drug metabolites, referred to as metabolites found only in humans or present at higher levels in humans than in animals, should be considered for safety assessment prior to drug approval. As such, the FDA recommends additional toxicity studies of these metabolites for safety concerns. However, the synthesis of sufficient quantities of drug metabolites, which often contain chiral centers, can be highly challenging using conventional chemical synthesis. Unfortunately, human P450s are poor biocatalysts for practical applications because of their instability, low turnover rate, and high cost.

Fortunately, an ideal biocatalyst for P450-catalyzed reactions is CYP102A1. Wild-type (WT) CYP102A1 catalyzes the regio- and stereoselective hydroxylation of long-chain fatty acids at a high turnover rate of >3000 min^−1^, the highest among all P450s [17,18,19]. Hydroxylation of long-chain fatty acids by CYP102A1 is highly coupled with a catalytic efficiency as high as 98%. Furthermore, recombinant CYP102A1 is highly expressed in bacteria and yeast at a yield of 0.5–1 g/L of the culture. CYP102A1 is a self-sufficient, highly active, and highly expressed biocatalyst for activating hydrocarbons.

The unique features of CYP102A1 have stimulated intense interest in engineering CYP102A1 to harness its catalytic prowess. Pioneered by Nobel Laureate Dr. Frances H. Arnold, a large repertoire of CYP102A1 variants has been generated by directed evolution and/or rational design to oxidize a plethora of non-natural substrates. Over 15,371 patents have been filed that utilize CYP102A1 variants in a wide range of applications. Here, we briefly review the recent advances in the understanding and application of CYP102A1, with a focus on protein engineering and design. This review is not intended to be comprehensive since several excellent reviews have been published in the past covering various aspects of CYP102A1 [6,18,20].

## 2. Structure and Function of CYP102A1

**Figure 2:**
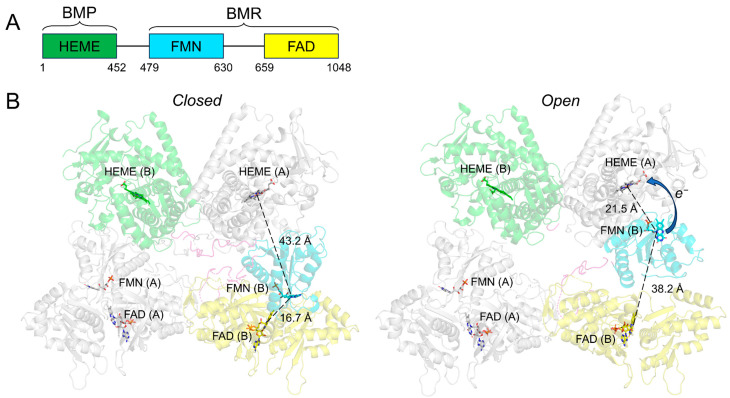
The primary structure architecture of CYP102A1 (**A**) and the “closed” and “open” conformations of the full-length CYP102A1 homodimer (**B**). The cryo-EM structure of full-length CYP102A1 was used to generate the models (EMDB: 20785 and 20786). Chain A is colored silver, while the three domains in chain B are colored using the same color scheme as (**A**). The chain in which the heme, FMN, and FAD cofactors are associated is shown in parentheses. The blue arrow indicates electron transfer from FMN to the heme. BMP, the catalytic heme domain; BMR, diflavinal reductase domain.

CYP102A1, found in *Bacillus megaterium* in the 1980s, is a soluble fusion form of the P450 enzyme, where its catalytic heme domain (BMP) and diflavinal reductase domain (BMR) are fused in a single peptide chain (Figure 2A). Both the BMP and BMR are highly homologous to the human P450 and POR, respectively. The catalytic properties of CYP102A1 can be attributed to its unique structural organization. The early work by Black and Martin found that CYP102A1 is not monomeric in a solution but rather oligomeric and likely a dimer [21]. It was later found that CYP102A1 is active as a homodimer at a salt concentration of >20 mM but inactive at a low salt concentration of ≤5 mM due to monomerization [22]. The salt-dependent dimerization of CYP102A1 points to an electrostatic nature responsible for dimerization.

**Figure 3:**
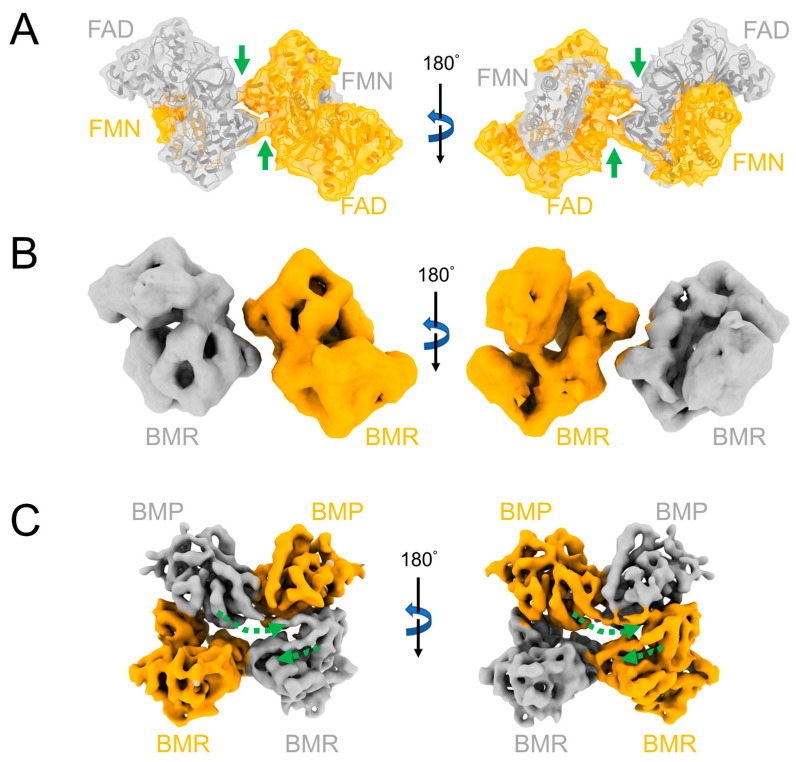
Comparison of the AlphaFold2 model for CYP102A1 homodimer with the cryo-EM models. The two chains of the homodimer are colored grey and orange, respectively. (**A**) The AlphaFold2 model showing the BMR domain. The backbone is shown in ribbons. The density map, displayed on a transparent surface, was generated at 7.6 Å using Molmap in ChimeraX. The green arrows show the densities connecting the FMN and FAD domains in the intermolecular-crossed complex. (**B**) Cryo-EM map of the full-length CYP102A1 homodimer at 7.6 Å showing the densities from the BMR domains only (EMDB: 20785). The contour threshold is 2.1 above the background. (**C**) Cryo-EM map of the Δ12 CYP102A1 homodimer at 6.3 Å showing both the BMP and BMR domains (EMDB: 21100). The dashed green arrows show the linker connecting the BMP and the FMN domains and the hinge connecting the FMN domain and FAD domain within the CYP102A1 monomer.

The inactive monomeric CYP102A1 raises an interesting question about the mechanism by which the electron is transferred from NADPH to the heme. Biochemical and site-directed mutagenesis studies have proposed two models: (1) intraflavin ET followed by interchain ET to the heme and (2) interflavin ET followed by intrachain ET to the heme [22,23]. The CYP102A1 homodimer structures determined by our group have provided strong evidence in favor of Model 1 [24]. As shown in Figure 2B, the CYP102A1 dimer exists in both “closed” and “open” conformations. The N-terminal BMP and C-terminal BMR domains are connected by a linker peptide. Intraflavin ET from the FAD to the FMN occurs in closed conformation due to the proximity of the FAD and FMN within the same BMR domain. The open conformation supports the interchain ET from the FMN to the heme since the rotation of the FMN subdomain shortens the FMN–heme distance from over 40 Å to ~21 Å [24] (Figure 2B). Consistent with mammalian P450s, the electron is delivered to the heme through the proximal side of the heme, where the thiolate ligand is located, as previously reported [25]. The structures of the individual domains and ET pathways of CYP102A1 are highly homologous to mammalian counterparts. Recently, an intermolecular-crossed complex model of the CYP102A1 homodimer was proposed by Urban and Pompon based on AlphaFold2 modeling [26]. In their model, as shown in Figure 3A, the FMN domain is dimerized with the FAD domain of the opposing monomer, which would favor interflavin ET and intrachain ET to the heme (i.e., Model 2). We thus compared the density map generated from their AlphaFold2 model with our cryo-EM density map. The simulated density map (7.6 Å) for the AlphaFold2 model shows the crossed-density connectivity between the FMN and FAD domains (Figure 3A). However, as shown in Figure 3B, the density connectivity is absent in our experimental cryo-EM map (7.6 Å) at an appropriate contour threshold level of >2.0 to remove background noises between the two BMR domains, suggesting that the two BMR domains are not interconnected. This is further supported by the cryo-EM map from a deletion of the CYP102A1 variant (Δ12) obtained at 6.3 Å (Figure 3C). The Δ12 variant lacks 12 amino acid residues in the linker region (amino acids 456–478). As a result of the deletion, the map was solved at a higher resolution and clearly shows the connectivity from the BMP domain to the FMN domain and then to the FAD domain within the same monomer.

Regardless of the mode of dimerization, the active site architecture of WT CYP102A1 is tailored to accommodate long-chain fatty acids through evolution. Thus, it shows poor activity toward non-natural substrates. For instance, the promiscuous activity toward non-natural substrates such as chlorzoxazone and coumarin is more than 100-fold lower than that of long-chain fatty acids [27]. Extensive efforts have been made in the past three decades to engineer CYP102A1 for the oxidation of non-natural substrates. Most of the efforts have focused on the modification of the catalytic BMP domain, especially residues lining the active site. In addition, many peripheral residues in the BMP domain have been reported to significantly affect the catalytic activity for reasons that are not well understood. It is also recognized that residues in the linker region and BMR domain may also affect the catalytic efficiency. We reported that shortening the linker enhanced the hydroxylation of omeprazole (OMP) by the CYP102A1 A82F variant, whereas others reported that mutations in the FAD domain enhanced coupling reactions [28]. Readers are referred to the excellent review by Whitehouse and coworkers for an extended list of sites mutated to engineer CYP102A1 [18].

## 3. Engineering and Design of CYP102A1

Protein engineering and design require modification of protein primary sequences to achieve desirable protein properties, such as catalytic activity, stability, regio- and stereoselectivity, etc. Many methods and strategies have been applied to engineer and design CYP102A1, generating novel applications.

### 3.1. Laboratory Directed Evolution (LDE)

LDE is the most widely used method to engineer CYP102A1 and many other protein targets. It involves the construction of mutant libraries, selection of desirable mutants by screening and triage, and validation with a purified variant protein. To construct the libraries, mutations are introduced by random mutagenesis, targeted mutagenesis, or recombination (family shuffling between homologs). Random mutagenesis utilizes error-prone DNA polymerase to induce mutations randomly in the cDNA sequence of CYP102A1, primarily in the BMP domains. Target mutagenesis can be carried out at selected sites to saturate the mutations. Mutation saturation at the selected sites with prior knowledge can greatly enhance the efficiency in obtaining mutants with desired properties. Recombination can generate chimeric enzymes using protein homologs (usually >70% of the sequence homology). Although LDE is highly efficient for constructing mutant libraries, the burden of success depends on the screening and triage of positive hits with the desired properties.

Using LDE, a large library of novel CYP102A1 variants have been identified that carry out the oxidation of a variety of non-natural substrates, including alkane, alkene, aromatics, pharmaceuticals, drugs, etc. WT CYP102A1 is not known to oxidize alkanes. By directed evolution of the BMP domain coupled with a rapid, reproducible colorimetric screening method, novel variants of CYP102A1 were found to oxidize octane to octanol [29]. After two generations of directed evolution, the turnover rate for the oxidation of octane increased by more than five-fold compared with that of WT CYP102A1. Additional rounds of LDE with modified screening methods permitted the selection of mutant 139-3, which turned out to be the fastest alkane hydroxylase known [30]. In addition, mutant 139-3 is also capable of oxidizing a range of alkenes, including styrene, benzene, cyclohexene, 1-hexene, and propylene [31]. LDE introduced a total of 11 mutations in mutant 139-3 (V78A, H138Y, T175I, V178I, A184V, H236Q, E252G, R255S, A290V, A195T, and L353V). Out of the 11 residues, V78A was the only residue in direct contact with the substrate. This raises intriguing questions about the roles of peripheral residues.

Another promising application of engineering CYP102A1 by LDE is found in the synthesis of human drug metabolites. The synthesis of sufficient quantities of human drug metabolites for preclinical bioactivity and toxicology evaluation can be challenging using conventional methods of chemical synthesis because human drug metabolites often contain chiral centers that require stringent control of regio- and/or stereoselectivity. As reported by Sawayama and coworkers [32], a panel of 120 CYP102A1 variants was selected by a combination of genetic diversification techniques, including random mutagenesis, targeted mutagenesis, and homolog recombination, to metabolize verapamil (a calcium channel blocker) and astemizole (antihistamine, withdrawn from the market in 1999). A subset of 43 variants was active in the metabolism of verapamil, generating all six human metabolites. For example, variant D6H10 converts 78% verapamil with a turnover rate of >1500 min^−1^, whereas variant 9-10A F87L converts 49% to a single metabolite with a 97% yield. A similar observation was made for astemizole, where a subset of 42 variants produced all seven human metabolites. It was reported by van Vugy-Lussenburg and coworkers that three generations of random mutagenesis resulted in a 200-fold increase in the turnover rate toward the drug substrate dextromethorphan and 3,4-methylenedioxymethylamphetamine [33]. Two of the mutations, F81I and E267V, which have great effects on activity, were not located in the active site. CYP102A1 variants generated by random mutagenesis were also found capable of producing chiral metabolites of simvastatin and lovastatin, two drugs prescribed to treat hyperlipidemia and hypercholesterolemia [34]. In humans, they are primarily metabolized by human CYP3A4/5 to produce several metabolites, including 6′β-hydroxy and exomethylene. The production of these metabolites can be recapitulated at high turnover rates by the M16 and M17 variants. Both variants contain multiple mutations in the BMP domain, including R47L, F81I, F87V, E143G, L188Q, and E267V in M16 and an extra mutation of E64G in M17. The same group also identified CYP102A1 variants by mutagenesis that metabolized OMP, a proton pump inhibitor used to treat gastric acid hypersecretion, to produce 5-hydroxy OMP; the reaction was, however, not stereoselective [35].

In addition to the BMP domain, LDE has also been applied to engineer the BMR domain. Unlike the oxidation of fatty acids by WT CYP102A1, a common problem with CYP102A1 variants in the oxidation of non-natural substrates is the low coupling efficiency; a large portion of consumed NADPH is non-productive, resulting in the formation of superoxide and hydrogen peroxide (Figure 1, the dashed arrows). Using a domain-based strategy where LDE was performed on each domain separately, followed by the combination of the best hits from each domain, Fasan and coworkers reported that novel variants, P450_PMO_R1 and P450_PMO_R2, exhibited native-like catalytic properties [28]. The two variants oxidized propane with >94% coupling efficiency. Mutations of P654K, D698G, and E1037G in the BMR domain contributed to the high coupling reaction.

LDE is, in general, effective in generating novel mutants with enhanced activity, provided that an effective screening method is available. Furthermore, it is sensitive to subtle changes in locations that are not well understood but exert great efforts on enzyme activity. However, the LDE workload can be demanding, with unpredictable outcomes. For a protein of 200 amino acids, it may be necessary to screen thousands of clones, which rapidly add up in multi-generations of evolution. As more mutant libraries are generated and the structure–function relationship of CYP102A1 is characterized in more detail, many residues that affect substrate selectivity and turnover have been recognized [36,37,38], which facilitates the rational design of CYP102A1 variants. LDE and rational design mutually benefit each other, facilitating the selection of desired mutants.

### 3.2. Rational Design

Based on prior knowledge of the structure–function relationship of CYP102A1, rational design can be efficient and cost-effective. Early work by Ost and coworkers rationally redesigned the substrate binding site of CYP102A1 to accommodate short-chain fatty acids (C4–C10) based on the crystal structure of WT CP102A1 in a complex with palmitoleate [39]. Mutation of the residues involved in palmitoleate binding, such as R47A/Y51F, L181K, and L181K/L75T, altered the substrate specificity to accommodate the short-chain fatty acids. The catalytic efficiency for hexanoate was increased by 15-fold in the L181K/L75T double mutant. Guided by the structural knowledge of CYP102A1, Dr. Munro’s group converted the WT CYP102A1 to a human CYP2C19-like enzyme by mutating a single residue (A82 → F) [36]. The A82F variant is capable of metabolizing OMP, a typical substrate of CYP2C19, to 5-hydroxy OMP. In humans, OMP is principally metabolized by CYP2C19 to produce 5-hydroxy OMP [40,41]. The catalytic efficiency (*k*_cat_/*K*_M_) of the A82F mutant for OMP hydroxylation is 78 µM^−1^·min^−1^. The OMP hydroxylase activity was further enhanced to 777 µM^−1^·min^−1^ in the A82F/F87V double mutant. Subsequent studies by the same group revealed that these variants also metabolize a range of proton pump inhibitors, including esomeprazole (*S*-OMP), lansoprazole, pantoprazole, and rabeprazole [42], providing new routes to the production of these metabolites. F87 is a key residue in the active site of CYP102A1; the mutation of F87 alters the binding and regioselectivity of fatty acids [18,37]. Thus, the enhancement in the hydroxylase activity by F87V can be rationalized. The role of A82F is intriguing. As documented by Butler and coworkers [36], A82 is a “gatekeeper” that controls conformation landscapes. The A82F mutation alters the landscapes in favor of the substrate-bound state. As we can see, knowledge-based design can be of great value in searching for novel variants for biotechnological applications.

### 3.3. Computational Protein Design (CPD)

Over the past decade, the availability of fast-speed computers and sophisticated algorithms for protein modeling has stimulated increasing attention to CPD. CPD offers researchers in silico tools to perform the redesign and de novo design of enzymes with more predictable outcomes and a higher probability of selecting the desired enzymes. It is a cost-effective approach, complementary to LDE since the time and cost spent for experimental testing are significantly reduced. CPD for enzyme design typically starts with a good understanding of the transition state (TS) or near-attack conformation (NAC) state for the substrate–enzyme complex. Then, an adequate ensemble of ligand conformers that approximate the TS/NAC state is generated in the active-site pocket of a given enzyme scaffold and used for enzyme sequence design. Next, the binding residues within the pocket are redesigned to enhance the ligand–enzyme binding affinity without destabilizing the enzyme’s active site. The designer sequences are usually triaged by an energy-based score function, which varies from one enzyme design tool to another. The designs can then be re-ranked and filtered by more computational analysis, such as molecular dynamics simulation. Regardless, the top-ranked designer enzymes need to be validated experimentally.

**Figure 4:**
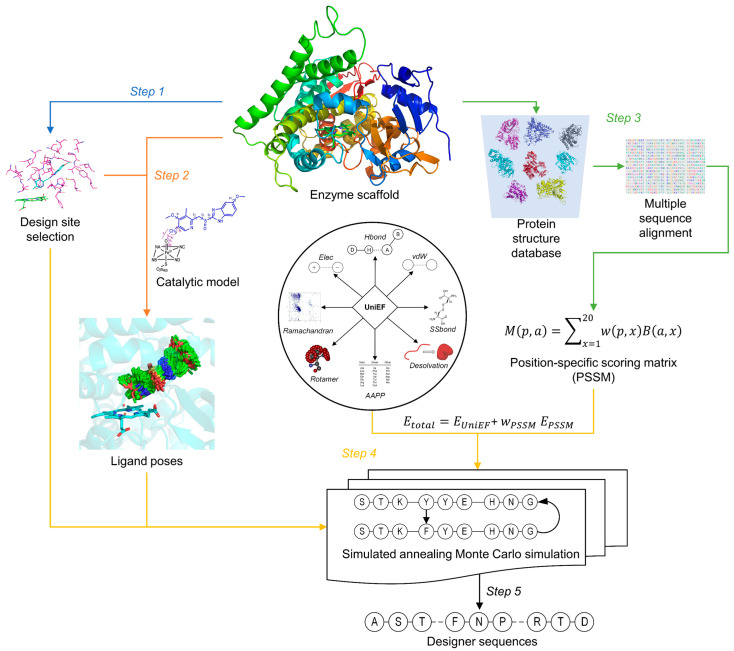
The UniDesign workflow for the computational enzyme design. The details of the five steps are described in the main text.

Recently, we presented UniDesign (https://github.com/tommyhuangthu/UniDesign, accessed on 15 May 2023) as a universal CPD framework for designing functional protein sequences. Its effectiveness has not only been demonstrated in protein–nucleic acid interaction design by accurately modeling the CRISPR/Cas9 PAM recognition [43] but also in small molecule-catalyzing enzyme design by redesigning CYP102A1 to improve its stereoselectivity for metabolizing OMP (in press). As illustrated in Figure 4, UniDesign consists of five steps: (1) define a suitable set of mutable design sites and another set of repackable sites based on the enzyme structure; (2) generate an ensemble of ligand conformers to mimic the TS/NAC state using a “grow-and-check” strategy, where the conformer generation needs to satisfy appropriate catalytic constraints defined by geometric parameters; (3) search for structure analogs to the enzyme scaffold to build an evolution-based position-specific scoring matrix (PSSM); (4) perform a simulated annealing Monte Carlo simulation to redesign/repack the chosen sites to optimize the weighted total energy of the enzyme–ligand system using a composite score function, which is a combination of the PSSM and a physics- and knowledge-based energy function (UniEF); and (5) rank and choose the top designs for experimental test.

Using UniDesign, we successfully redesigned CYP102A1 variants that hydroxylate *R*-OMP with high regio- and stereoselectivity. As aforementioned, Dr. Munro’s group reported that CYP102A1 mutants A82F and F87V and the double mutant (A82F/F87V) hydroxylate OMP to produce 5-hydroxy OMP [36,42]. Furthermore, a triple variant (R47L/F87V/L188Q) was reported to hydroxylate OMP to 5-hydroxy OMP [44]. However, these variants do not show stereoselectivity for *R*-OMP over *S*-OMP. OMP is administered as a racemate (50/50) consisting of *R*- and *S*-OMP. *R*-OMP is efficiently metabolized by CYP2C19, while *S*-OMP is slowly metabolized by CYP3A4 [41]. Differentiating the enantiomers would construct a truly 2C19-like CYP102A1 variant. According to the UniDesign binding energy for the two enantiomers, we selected three variants (UD1, UD2, and UD3) for experimentation. All three variants contain a triple mutant (A82F/F87V/L188Q) and one additional mutation of L75I in UD1, A264G in UD2, and A328V in UD3. Detailed characterization showed that all three variants hydroxylate *R*-OMP to produce 5-hydroxy OMP with high turnover rates; in marked contrast, they all exhibit low turnover rates for *S*-OMP. The enantiomeric excess (e.e.) values are 92, 87, and 70% for UD1, UD2, and UD3, respectively, a marked improvement over the previously reported CYP102A1 variants for OMP oxidation. The results demonstrate the effectiveness of UniDesign for engineering enzyme stereoselectivity.

In another study, the Rosetta CPD approach has been recently applied to CYP105AS1 to invert the stereoselectivity for the production of the cholesterol-lowering drug pravastatin [45]. CYP105AS1 from *Amycolatopsis orientalis* hydroxylates compactin (also known as mevastatin in the statin class) to 6-epi-pravastatin. To produce pravastatin, the stereoselectivity for the hydroxylation of compactin must be inverted from a pro-*R* to a pro-*S* reaction. LDE was initially adopted to engineer the stereoselectivity of CYP105AS1. More than 15,000 clones were screened and triaged in three generations of LDE. One variant carrying five mutations, referred to as P450_Prava_, was selected to produce pravastatin as a major product [45,46]. To further increase the e.e. value, Ashworth and coworkers employed the CoupledMoves protocol in Rosetta to design new variants from P450_Prava_. The advantage of the CoupledMoves protocol is the inclusion of protein plasticity in Rosetta, which generates an ensemble of mutant configurations. Short molecular dynamics simulations were performed to eliminate unwanted variants. In the initial round of searching, 14 positions in the active site were chosen based on the alignment analysis to homologous sequences in UniProt. As a result, 9052 low-energy designer sequences were generated, and eight out of the fourteen chosen positions showed credibility for modifying stereoselectivity to the pro-*S* pathway. Among them, P450_Prava_ T95F and P450_Prava_ V180M produced a roughly equal ratio of pravastatin to 6-epi-pravastatin, around 95:5. Rosetta modeling suggested that T95F and V180M were compatible, and their combination (P450_Prava_ T95F/V180M) produced nearly 100% pravastatin without detection of any unwanted side products.

Li and coworkers employed an integrated approach, combining CPD, evolutionary information, and experimental data-driven optimization to redesign a plant P450 enzyme CYP87D20 for the production of mogrol [47]. The plant family Cucurbitaceae biosynthesizes cucurbitacins, bitter taste compounds, and mogrosides, potent natural sweeteners, from the same cucurbitane backbone. In cucumber, the biosynthesis of cucurbitacin C is catalyzed by CYP87D20 to oxidize cucurbitadienol at C11 to form a keto product, 11C-cucurbitadienol, and then at C25 to form a hydroxylated product, 11C-20H-cucurbitadienol. In the first step toward a new metabolic pathway to biosynthesize mogrol, the authors redesigned CYP87D20 to carry out a single hydroxylation at C11 to produce 11H-cucurbitadienol. Due to the lack of crystal structures of plant CYP enzymes, a homology model was constructed using Rosetta, followed by docking cucurbitadienol into the active site. All side-chain and backbone atoms with 6 Å of the ligand were analyzed using evolutionary information by both a PSSM and their co-evolutionary couplings. In the first round of design, 24 positions with positive PSSM values were identified, resulting in 30 designs that were experimentally evaluated. The results showed that E286 and F112 were key residues for the discrimination between the hydroxylation and ketone formations at C11 of the substrate. This piece of information was then fed into the subsequent rounds of optimization to further improve specificity and activity. The authors systematically generated all protein variants in silico, where >27,000 designs were clustered and filtered. Several design variants, such as L109/F113L/E286A, showed high selectivity to produce 11H-cucurbitadienol.

The CPD methodology is clearly cost-effective in surveying a large protein sequence space for the intended properties. However, the outcome is weighed on an energy-based score function and knowledge of the enzyme–ligand complex. Many factors affect enzyme–ligand interactions, but their effects may not be fully accounted for. For instance, the effects of peripheral residues and conformational dynamics on enzyme activity are difficult to quantify in terms of energy contribution. If no experimental structure is available, CPD will depend on homology models that need validation.

### 3.4. Machine Learning (ML)

**Figure 5:**
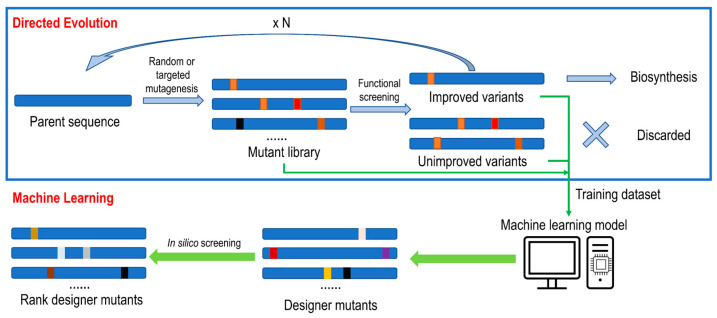
Directed evolution and machine learning workflows for enzyme engineering. For directed evolution, random or targeted mutations were introduced into parent sequences, generating libraries of mutants. After screening for function, improved variants will be selected for the next round of mutagenesis or used for biosynthetic purposes, and unimproved variants will be discarded. For machine learning models, both improved and unimproved variants will be for modeling training and testing. The machine learning-designed sequences can undergo further in silico screening and ranking before experimentation.

The “holy grail” for protein design and engineering is a fully automated process with little human intervention. The success of the Google AlphaFold project propels innovations in this direction. Through deep learning, AlphaFold2 employs a neural network with repeated layers to predict protein 3D structures from input primary sequences, resulting in astonishingly accurate predictions [48]. The goal to predict protein function seems increasingly plausible with the development of machine learning (ML) algorithms and available big data. Over the past three decades, the LDE of CYP102A1 has already generated a large amount of data, but only a tiny portion of which is deemed useful for the selection of positive clones, while unimproved and inactive mutants toward specific substrates are discarded. However, for ML models that are data-driven, both functionally improved and unimproved/inactive mutants can be useful for model training and refinement, as illustrated in Figure 5. LDE-, CPD-, and ML-based enzyme design approaches are mutually beneficial to each other, not exclusive [49,50].

There are multiple types of algorithms with different specialties having the same goal of discovering the patterns within provided datasets. The most straightforward model is linear regression, which directly correlates the input with the output. Linear algorithms are fundamental ML models typically used to test the suitability of datasets for training purposes. In most cases, a well-prepared dataset is split into at least two parts, training and testing (and sometimes having one more group for validation) [50,51,52]. There are many other advanced ML models, including regression trees, neural networking, and the Gaussian process (GP) [53,54,55]. It is not tenable to assert the superiority or inferiority of one model over another, as all models are inherently flawed. However, sometimes, some are useful.

ML algorithms are classified into supervised and unsupervised, depending on the labeling of the datasets. Supervised ML models are usually adopted for enzyme function predictions due to the presence of labeled enzyme properties, including the catalytic parameters, solubility, and thermostability. They can efficiently discover the patterns between characterized features and labels, forming a decision boundary to predict the properties of the unlabeled or designer inputs [52]. Unsupervised ML models are usually used to analyze unlabeled data so that some unknown sequence–function relationships can be found. However, it can be harder to test the accuracy of unsupervised ML models because unlabeled data may not provide good standards (decision boundaries) for classification.

In 2013, Romero and coworkers utilized the GP, a Bayesian learning technique, to navigate how protein sequence maps function, termed the “fitness landscape”, and they demonstrated that the fitness landscape could be inferred from experimental data with high accuracy [56]. Various protein sequence properties, such as the functional status, thermostability, enzyme activity, and ligand binding affinity, can be modeled using the GP. Initially, the GP model was trained on a set of 242 experimental T_50_ data (the temperature at which half of the protein is irreversibly inactivated in 10 min) from a diverse set of chimeric P450s whose sequences were generated by combining eight sequence fragments from the heme domains of CYP102A1, CYP102A2, and CYP102A3 (the three isoforms share an ~65% pairwise sequence identity). The trained GP model showed excellent predictive power with a cross-validated Pearson correlation coefficient of 0.95 and a mean absolute error of 1.4 °C. The GP model outperformed a linear regression model weighed on individual sequence fragments. Furthermore, training directly on the experimental data allows the GP model to capture numerous implicit factors, most of which are unknown, and determine whether a sequence will encode a functional P450 enzyme. The trained model predicted 20 sequences for functional enzymes, 17 out of which were experimentally confirmed. Building on this set of sequences, the second-round design produced nine out of ten sequences. Next, a total of 29 functional enzymes were expressed and characterized. The experimental data were used in the GP model to predict the enzyme activity and ligand binding affinity. After a few rounds of optimization, the GP model predicted sequences encoding for highly thermostable enzymes. The most stable enzyme, EXPc5, had a T_50_ of 69.5 °C, higher than any previously identified variants through directed evolution [56,57].

Although the application of ML models to P450 enzymes is scarce, the number of ML applications to other enzyme targets has mushroomed [51,58]. Fox and coworkers used a partial least-square regression algorithm to identify beneficial mutants in a bacterial halohydrin dehalogenase, leading to an ~4000-fold increase in volumetric productivity [59]. This met the criteria for the commercially relevant process to produce atorvastatin. Liao and coworkers combined high-throughput gene synthesis and ML-based algorithms to engineer proteinase K [60]. Through two cycles of training and testing, a 20-fold increase in proteinase K activity was achieved after testing only 95 variants. Du and coworkers used ML to assist in the design of new antioxidants [61]. Han et al. reported an ML application to improve protein solubility and activity [62].

### 3.5. Chemical Engineering

**Figure 6:**
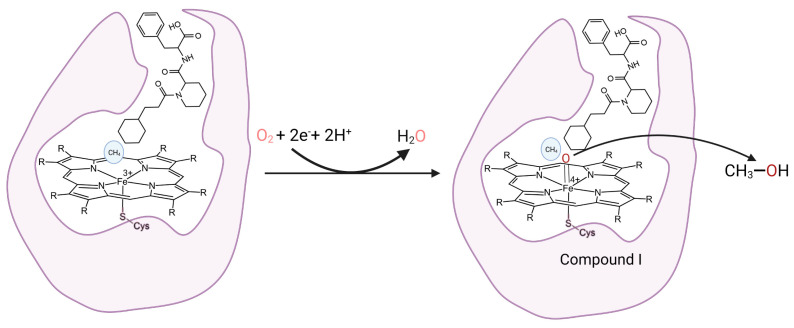
Illustration of the decoy molecule system for oxidation of small molecule substrate by WT CYP102A1. The small light-blue circle represents the small molecule substrate, methane, whereas Cys-S represents the thiolate ligand to the heme iron. The decoy molecule (3CHPA-Pip-Phe) is positioned at the entrance of the substrate’s binding pocket, facilitating the binding of methane.

The aforementioned strategies are aimed at tuning the protein properties to fit substrates of interest. Conversely, chemical engineering optimizes substrate oxidation not by altering the enzyme but rather by fitting the substrate of interest into the active site of the WT CYP102A1 with a decoy molecule system [63,64] (Figure 6). Taking advantage of the high oxidizing potential of Compound I, decoy molecules facilitate the generation of Compound I and the positioning of the substrate close to the heme iron for oxidation. Assisted with perfluorinated carboxylic acids as decoy molecules, WT CYP102A1 is capable of oxidizing gaseous alkanes, such as propane and butane, as well as benzene and toluene [64,65]. Recently, WT CYP102A1 was reported to oxidize small gaseous methane to methanol at room temperature at 10 MPa [66]. It is notoriously difficult to functionalize methane; the small size of methane does not fit well into the active site of CYP102A1. Using the decoy molecule (3CHPA-Pip-Phe) that mimics N-palmitoylglycine, the authors were able to sequester methane in the proximity of the heme iron for oxidation.

## 4. Concluding Remarks

Protein design and engineering have transformed CYP102A1 from a fatty acid hydroxylase with high specificity to a highly versatile biocatalyst capable of oxidizing a wide range of non-natural substrates. The utility of CYP102A1 variants has extended to many areas of applications, such as drug discovery, biotechnology, biosynthesis, bioremediation, and beyond. LDE and structured-based CPD methods play a major role in the design and engineering of CYP102A1 for this remarkable transformation. LDE is relatively straightforward to implement, but the outcome depends on rapid, reproducible screening methods, and the workload can be daunting. CPD is cost-effective but requires solid structure–function modeling to effectively design functional variants. Thanks to the functional and structural characterization of the WT and variants of CYP102A1, a wealth of information is already available, which undoubtedly facilitates the CPD approaches. Nonetheless, the factors that affect enzyme activity are complex and not fully understood. Moreover, the protein sequence space is vast, and the functional variant constitutes only a tiny fraction of the sequence space. Thus, the energy-based score function employed by CPD may not fully account for all these factors. The structure–function relationship of CYP102A1 merits further investigation, and we need a holistic approach to understand the molecular machinery of CYP102A1. One of the main challenges for the use of CYP102A1 on the preparative scale is to improve the coupling efficiency, which requires careful consideration of the interactions between the BMP and BMR domains. The cryo-EM structures of the full-length CYP102A1 homodimer show that the CYP102A1 homodimer is highly dynamic, and the ET from the BMR domain to the heme domain involves significant conformational changes, contributing to the overall coupling. The more information available about the structure and function of CYP102A1 mutants, the better ML models can be constructed to predict new variants with enhanced enzyme properties. The use of ML to design and engineer enzymes is a new field with tremendous potential. The success of ML depends on experimental big data and specific algorithms to augment its predictive power. ML for CYP102A1 engineering is in its infancy, with one successful application to improve the thermostability of chimeric P450 enzymes, but the outcome is astonishing. We anticipate that more ML applications to CYP102A1 will emerge in the future.

## Data Availability

Not applicable.
